# Metabolic and transcriptional profiling reveals pyruvate dehydrogenase kinase 4 as a mediator of epithelial-mesenchymal transition and drug resistance in tumor cells

**DOI:** 10.1186/2049-3002-2-20

**Published:** 2014-11-03

**Authors:** Yuting Sun, Anneleen Daemen, Georgia Hatzivassiliou, David Arnott, Catherine Wilson, Guanglei Zhuang, Min Gao, Peter Liu, Aaron Boudreau, Leisa Johnson, Jeff Settleman

**Affiliations:** Department of Discovery Oncology, Genentech Inc, 1 DNA Way, 94080 South San Francisco, CA USA; Department of Bioinformatics, Genentech Inc, 1 DNA Way, 94080 South San Francisco, CA USA; Department of Translational Oncology, Genentech Inc, 1 DNA Way, 94080 South San Francisco, CA USA; Department of Protein Chemistry, Genentech Inc, 1 DNA Way, 94080 South San Francisco, CA USA

**Keywords:** Tumor metabolism, EMT, Drug resistance, Pyruvate dehydrogenase kinase

## Abstract

**Background:**

Accumulating preclinical and clinical evidence implicates epithelial-mesenchymal transition (EMT) in acquired resistance to anticancer drugs; however, mechanisms by which the mesenchymal state determines drug resistance remain unknown.

**Results:**

To explore a potential role for altered cellular metabolism in EMT and associated drug resistance, we analyzed the metabolome and transcriptome of three lung cancer cell lines that were rendered drug resistant following experimental induction of EMT. This analysis revealed evidence of metabolic rewiring during EMT that diverts glucose to the TCA cycle. Such rewiring was at least partially mediated by the reduced expression of pyruvate dehydrogenase kinase 4 (PDK4), which serves as a gatekeeper of the TCA cycle by inactivating pyruvate dehydrogenase (PDH). Overexpression of PDK4 partially blocked TGFβ-induced EMT; conversely, PDK4 inhibition via RNAi-mediated knockdown was sufficient to drive EMT and promoted erlotinib resistance in *EGFR* mutant lung cancer cells. We identified a novel interaction between PDK4 and apoptosis-inducing factor (AIF), an inner mitochondrial protein that appears to play a role in mediating this resistance. In addition, analysis of human tumor samples revealed *PDK4*-low as a predictor of poor prognosis in lung cancer and that *PDK4* expression is dramatically downregulated in most tumor types.

**Conclusions:**

Together, these findings implicate PDK4 as a critical metabolic regulator of EMT and associated drug resistance.

**Electronic supplementary material:**

The online version of this article (doi:10.1186/2049-3002-2-20) contains supplementary material, which is available to authorized users.

## Background

Acquired drug resistance has emerged as a major challenge to effective cancer therapy. Tumor-targeted therapies, such as anti-EGFR treatments for lung cancer and anti-HER2 treatments for breast cancer, are highly effective in disease management in biomarker-defined patient populations. However, the vast majority of these treated patients’ cancers ultimately become drug resistant due to the presence of a subpopulation(s) of malignant cells that survive the therapy and repopulate the relapsed tumor, thereby limiting the long-term effectiveness of these targeted agents [1].

Epithelial-mesenchymal transition (EMT) is a biological process during which epithelial cells lose the expression of epithelial markers and gain the expression of mesenchymal markers, imparting cells with distinct morphological, migratory and other properties. EMT has frequently been observed in drug-resistant cancer cells in both preclinical models and clinical samples [2], including resistance to anti-EGFR therapy in lung cancer [3, 4], resistance to androgen-deprivation therapy in prostate cancer [5], and resistance to chemotherapy in breast cancer [6]. In addition to its role in drug resistance, EMT also converts epithelial cancer cells into a more metastatic and “stem-like” state [7].

Altered metabolism has been recognized as a hallmark of cancer [8]. Tumor cells need to consume more glucose and glutamine to satisfy the energy demand and biosynthesis requirement for rapid proliferation. Although considered as auxiliary to tumorigenesis for many years, recent studies have revealed oncogenic mutations in the core metabolism network that could potentially drive tumor initiation and progression [9]. In addition, accumulating evidence has revealed that altered metabolism is essential for activated oncogenes and inactivated tumor suppressors to drive malignant transformation [10]. Considering the profound changes in cellular metabolism in cancer, we hypothesized that tumors with acquired drug resistance might resort to alternative mechanisms to fuel their growth and survival under drug treatment. In this study, we report a diversion of glucose metabolism towards the TCA cycle during EMT in cancer cells. We also provide evidence that pyruvate dehydrogenase kinase 4 (PDK4) is a critical regulator of EMT and associated drug resistance.

## Methods

### Antibodies and other reagents

Antibodies used are listed as follows: E-cadherin (Cell Signaling, 24E10), N-cadherin (Cell Signaling, no. 4061); Vimentin (Cell Signaling, D21H3), GAPDH (Cell Signaling, 14C10), Zeb1 (Cell Signaling, D80D3), Snail (Cell Signaling, C15D3), VDAC (Cell Signaling, D73D12), apoptosis-inducing factor (AIF) (Cell Signaling, D39D2), and FLAG (Sigma, M2). PDK4 polyclonal antibody was generated by immunizing rabbits against a peptide corresponding to amino acids 264–277 of human PDK4 (VEHQENQPSLTPIE) (Yenzyme).

### Cell culture

Unless otherwise specified, all cell lines were cultured in RPMI1640 (Gibco) containing 2 g/l sodium bicarbonate, 10% FBS, 2 mM P/S, and 4 mM glutamine (Gibco). To generate the TGFβ-induced mesenchymal cells, cells were cultured in the presence of 2 ng/ml TGFβ (Cell signaling) for 2 to 5 weeks. A549 cells were cultured with TGFβ for 2 to 3 weeks. HCC827 and NCI-H358 cells were cultured with TGFβ for 3 to 5 weeks prior to experiments. Fresh media containing TGFβ was replenished every 3–4 days. The day before the experiment, cells were re-seeded in the appropriate plates without TGFβ. Cell lines are maintained by a core facility at Genentech that routinely uses STR fingerprinting to verify cell line identity.

### Data analysis

Unless otherwise specified, all data plotting and statistical analysis was performed using Prism Graph Pad 5.0, and the error bars represent SEM. Student's *t* test was used to assess the statistical significance of the differences between groups (two-tail *p value <0.05; two-tail **p value <0.01.

Survival analyses were performed with the Kaplan-Meier method and Cox proportional-hazard model. Results across the three data sets (GSE42127, GSE8894, and GSE3141) were combined in a meta-analysis, using the R package meta. The overall combined estimate of the hazard ratio was obtained from their values and standard errors in the individual data sets.

*PDK4* expression data in normal lung, lung adenocarcinoma and squamous cell carcinoma of the lung was generated from TCGA RNA-seq data, which was obtained from the Cancer Genomics Hub at UC Santa Cruz and preprocessed and aligned with HTSeqGenie [11]. *PDK4* expression data in multiple cancer indications was from the Gene Logic database of microarray data using GeneChip human genome U133 Plus 2.0 array (Affymetrix). Expression summary values for all probe sets were calculated using the RMA algorithm as implemented in the affymetrix package from Bioconductor.

### Global metabolomic profiling

The parental and TGFβ-induced mesenchymal cells were rinsed with PBS, scraped in PBS, and spun down. The cell pellets were snap-frozen and submitted to Metabolon Inc for global metabolomic analysis [12]. Briefly, a combination of GC-MS and LC-MS methods were used, and each metabolite amount was normalized to total protein amount of the individual cell pellets. Each sample consisted of cells collected from two 15-cm plates at approximately 60% confluence, and each condition included five replicates.

### Glycolysis/OXPHOS ratio measurement

Real-time Glycolysis/OXPHOS rate was measured using the Seahorse metabolic analyzer, following manufacturer's protocols. Briefly, cells were plated in six replicates in 96-well Seahorse assay plates. The seeding cell numbers were adjusted based on cell growth rate, with the goal to reach similar cell density at the time of the real-time measurement. The next day, cells were washed twice and incubated in 100 μl of modified RPMI1640 growth media for 2 h. The modified RPMI1640 growth media did not contain sodium bicarbonate, and contained dialyzed FBS (Gibco) instead of standard FBS. Proton production rate (PPR) and oxygen consumption rate (OCR) were recorded.

### Mass isotopologue distribution analysis using C-13 stable isotopes

Cells were plated in a 15-cm plate overnight, and then switched to tracing media. The tracing media was based on standard RPMI1640 growth media containing 10% dialyzed FBS, with either glutamine substituted by ^13^C-U5-glutamine or glucose substituted by ^13^C-U6-glucose (Cambridge Isotope). After being cultured in the tracing media for 24 h, cells were harvested and processed for mass spectrometry. A detailed description of the mass spectrometry analysis is provided in ‘Extended Methods.’

### Microarray gene expression analysis

Gene expression profiling comparing TGFβ-treated mesenchymal cells and corresponding parental cells was performed using GeneChip human genome U133 Plus 2.0 array (Affymetrix), following standard protocols. Data were normalized using the R package RMA from Bioconductor and analyzed with the R limma package. The expression microarray data has been deposited in the Gene Expression Omnibus (GEO) database under accession number GSE49644.

### Extended methods

Description of additional methods is provided in Additional file 1.

## Results

### Experimentally-induced EMT in lung cancer cell lines is associated with metabolic reprogramming

**Figure 1:**
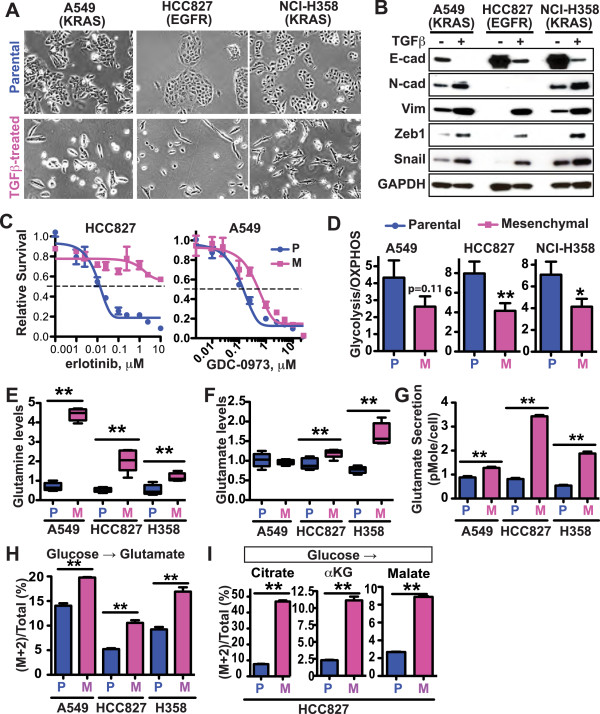
**Metabolic changes in three NSCLC cell lines upon TGFβ-induced EMT.** A549, HCC827, and NCI-H358 lung cancer cells were cultured in the presence of 2 ng/ml TGFβ for 2 to 5 weeks to induce EMT. The following aspects of both the parental (P) and mesenchymal (M) cells were characterized. **(A)** Morphological changes of cells. **(B)** The expression of the epithelial marker (E-cadherin) and mesenchymal markers (N-cadherin, Vimentin, Zeb1, and Snail). **(C)** Erlotinib sensitivity of HCC827 and GDC-0973 sensitivity of A549 parental and mesenchymal cells. The cells were treated with the EGFR kinase inhibitor erlotinib or MEK inhibitor GDC-0973 for 3 days, and viability was measured using a CellTiter-Glo assay. **(D)** Glycolysis/OXPHOS ratio, defined by PPR/OCR and measured using the Seahorse metabolic analyzer. Average results from three to four independent experiments are shown. **(E, F)** Cellular glutamine **(E)** and glutamate **(F)** concentrations as measured by mass spectrometry. Each data point is from five separate biological samples generated at the same time. The *boxes* represent 10–90 percentile. **(G)** Glutamate secretion per cell during 24 h. Average of data from six wells in one experiment, which is representative of three independent experiments, is shown. **(H, I)** Cells were incubated with growth media containing ^13^C-U-glucose overnight, and then subjected to LC-MS analysis. **(H)** Glucose to glutamate contribution was plotted based on the percentage of (M + 2) glutamate in the total glutamate pool. **(I)** Glucose to TCA cycle contribution was plotted based on the percentages of (M + 2) citrate, (M + 2) α-ketoglutarate and (M + 2) malate in each individual metabolite’s total pool. For all panels, data are plotted as mean ± SEM. *p < 0.05; **p < 0.01, unless otherwise specified.

Human cancer cell lines provide essential models for dissecting fundamental mechanisms in tumor biology. We modeled EMT in cultured cancer cells using TGFβ treatment since TGFβ robustly induces EMT in many epithelial cell line models, and physiologically, hyperactivation of TGFβ signaling has been shown to be associated with the mesenchymal phenotype and cancer drug resistance [13, 14]. To identify EMT-associated changes in cancer cell biology that are not restricted to one specific genetic background, we examined three different human non-small cell lung cancer (NSCLC) cell lines—A549 (KRAS^G12S^-driven), HCC827 (EGFR^ΔE746-A750^-driven), and NCI-H358 (KRAS^G12C^-driven). We cultured cells with continuous exposure to TGFβ for 3 weeks and observed dramatic morphological transformation—the cells changed from displaying a compact epithelial morphology with obvious cell-cell contacts to a fibroblast-like morphology with scattered spindle shapes (Figure 1A). Consistent with the morphological changes observed in the TGFβ-treated cells, we detected decreased expression of the epithelial marker E-cadherin as well as increased expression of the mesenchymal markers N-cadherin and Vimentin and the EMT-associated transcription factors Zeb1 and Snail (Figure 1B).

Consistent with previous studies implicating EMT in drug resistance, the derived mesenchymal cells became significantly less sensitive to targeted therapies. The mutant EGFR-driven HCC827 parental cells were very sensitive to erlotinib, whereas the TGFβ-induced mesenchymal derivatives had lost their EGFR ‘addiction’ and became largely resistant to erlotinib (Figure 1C). Likewise, the mesenchymal derivatives of A549 and NCI-H358 cells became significantly less sensitive to the MEK inhibitor GDC-0973 (Figure 1C) and KRAS inhibition via RNAi (see Figure three F in reference [15]), respectively. Taken together, these findings demonstrate an EMT upon chronic TGFβ treatment of these ‘oncogene-addicted’ lung cancer cells and provide a model system for studying the role of EMT in mediating drug resistance *in vitro.*

In the course of culturing these cells, we observed slower acidification of the media in the mesenchymal derivatives relative to the corresponding untreated parental cell lines, suggesting a possible metabolic rewiring event during EMT. A switch from oxidative phosphorylation (OXPHOS) to aerobic glycolysis, the so-called Warburg effect, is a key feature of many cancer cells [16]. Whereas glycolysis is a relatively rapid mechanism by which cells can produce ATP, OXPHOS is much more energy efficient as it produces more ATP per glucose molecule. We therefore measured real-time glycolysis and OXPHOS levels in these cells and observed a consistent decrease in the glycolysis/OXPHOS ratio upon EMT in all three cell lines (Figure 1D). Moreover, this glycolysis-to-OXPHOS switch was not restricted to TGFβ-induced EMT: EGFR-mutant HCC4006 cells that had been selected for resistance to erlotinib and which gained mesenchymal features [17], similarly, exhibited a decreased glycolysis/OXPHOS ratio relative to their parental, erlotinib-sensitive counterpart (Additional file 2: Figure S1A). These collective data reveal a metabolic remodeling process commensurate with EMT.

### Metabolomic profiling reveals amino acid accumulation associated with EMT

The observed glycolysis-to-OXPHOS switch during EMT prompted us to examine the global metabolomic profiles of these cells before and after EMT using mass spectrometry (Additional file 3: Table S1). Using an absolute fold-change >2 and a *p* value <0.05 as the cutoff, we observed only ten metabolites (out of approximately 400 known metabolites measured) that showed consistently significant changes during EMT across all three cell lines (Additional file 2: Figure S1B). Among them, five metabolites—glutamine, tryptophan, reduced glutathione, 4-guanidinobutanoate, and cysteinylglycine—belong to the amino acid or peptide category. We further examined the levels of the 20 standard amino acids before and after EMT and found an overall increase in amino acid levels in the mesenchymal cells in all three models (Additional file 2: Figure S1C).

We were particularly intrigued by the observed changes in glutamine (Figure 1E) and glutamate (Figure 1F) due to the direct interconversion between these two metabolites. The accumulation of intracellular glutamate was not due to decreased glutamate efflux. In fact, we observed a significant (*p* < 0.01) increase in glutamate secretion across all three mesenchymal cell populations relative to their corresponding parental controls (Figure 1G). Notably, although the TGFβ-treated cells grew about two fold slower than their parental counterparts (Additional file 2: Figure S1D), it is unlikely that the observed glutamate accumulation is simply a consequence of reduced proliferation rate since metabolomic profiling of the NCI-60 cell line panel did not reveal any association between glutamate accumulation and cell proliferation rate [18]. Together, these findings demonstrate glutamate accumulation associated with the conversion of epithelial cancer cells to a mesenchymal state.

### Glucose diversion to the TCA cycle contributes to increased intracellular glutamate in mesenchymal cells

**Figure 2:**
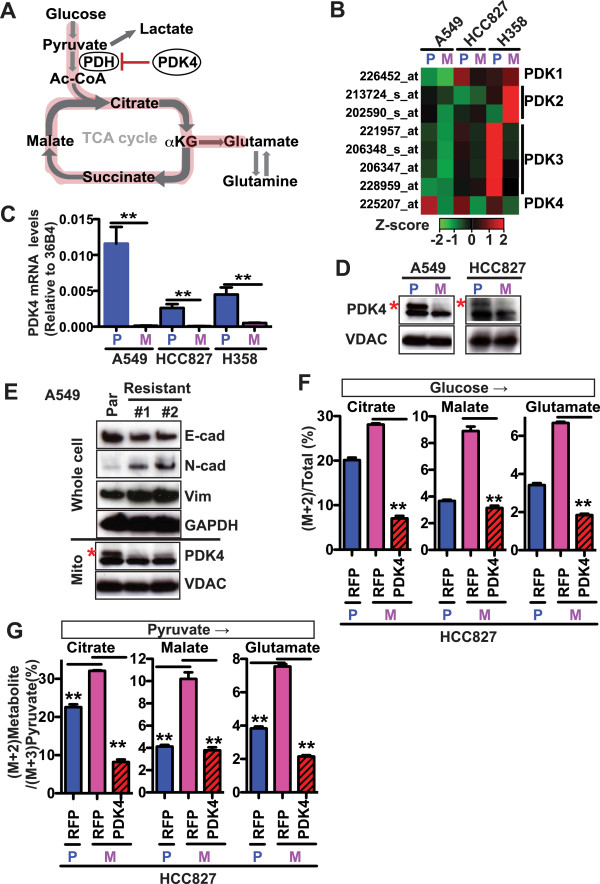
**PDK4 downregulation is observed in mesenchymal cells, and mediates metabolic rewiring. (A)** A schematic representation of the metabolic rewiring observed in TGFβ-induced mesenchymal cells. *Red highlight* indicates increased flux from glucose to the TCA cycle and then to glutamate in the mesenchymal cells. **(B)** Heatmap based on microarray gene expression data showing the mRNA levels of *PDK1-PDK4* in the parental cells (*P*) and mesenchymal derivatives (*M*). **(C)** Quantitative RT-PCR showing *PDK4* mRNA levels in parental and mesenchymal cells. In **B** and **C**, data shown is the average of three separate biological samples generated at the same time. **(D)** Western blots with the mitochondria showing the PDK4 protein levels in parental and mesenchymal derivatives. VDAC was used as the protein loading control. The *red asterisk* denotes the upper band that specifically corresponds to PDK4. **(E)** Immunoblotting for PDK4 and EMT markers in A549 parental (*Par*) and resistant cells. The resistant clones were generated by culturing A549 cells in the presence of 0.5 μM GDC-0973 and 0.2 μM GDC-0941 for 2 months. Data from two independent clones is shown. **(F, G)** HCC827 parental and mesenchymal cells were infected with lentivirus expressing RFP or PDK4 with a GFP reporter. The GFP-positive cells were selected by FACS, cultured in growth media containing ^13^C-U-glucose overnight, and then subjected to LC-MS analysis. **(F)** Glucose to TCA cycle/glutamate contributions were plotted based on the percentages of (M + 2) citrate, (M + 2) malate and (M + 2) glutamate in each metabolite’s respective total pool. **(G)** Pyruvate to glutamate contribution was plotted based on the ratio of glucose → glutamate (the percentage of (M + 2) glutamate in total glutamate pool) over glucose → pyruvate (the percentage of (M + 3) pyruvate in total pyruvate pool). Pyruvate to citrate or malate contribution was similarly plotted. Data are plotted as mean ± SEM. **p < 0.01.

Next, we addressed the mechanism underlying the observed glutamate accumulation in the mesenchymal cells. The likely potential carbon sources are either glutamine or glucose. Glutamine can be converted directly to glutamate via deamination. Glucose, in contrast, can be converted to glutamate through a multi-step process [10]. During the early steps of glycolysis, glucose is first converted to pyruvate and then to acetyl-CoA, which directly enters the TCA cycle. Alpha-ketoglutarate, a critical intermediate metabolite in the TCA cycle, serves as the backbone for the synthesis of many amino acids, including glutamate. To assess the glucose and glutamine contributions to glutamate and glutathione, we performed mass isotopologue distribution analysis (MIDA) [19]. We cultured the epithelial and mesenchymal cells with C-13 uniformly labeled glucose or glutamine, and quantified the incorporation of C-13 label into glutamate using liquid column-mass spectrometry (LC/MS). Although glutamine was still a major source of the glutamate pool in both the parental and mesenchymal cells, we found significantly decreased percentages of glutamine-derived glutamate (Additional file 2: Figure S1E) and corresponding increased percentages of glucose-derived glutamate (Figure 1H) in all three mesenchymal models compared to their corresponding parental cells. Importantly, we also observed significantly increased percentages of glucose-derived TCA cycle metabolites, including citrate (the first metabolite in the TCA cycle), α-ketoglutarate (αKG, a critical precursor for amino acid synthesis), and malate (Figure 1I), consistent with the notion that the TCA cycle contributes to the observed glutamate accumulation during EMT. Collectively, these data demonstrate a diversion of glucose to the TCA cycle → glutamate axis in the mesenchymal cells (Figure 2A).

### PDK4 is downregulated in mesenchymal cells and regulates glucose contribution to the TCA cycle

To further explore the molecular mechanisms underlying the observed metabolic rewiring upon EMT, we compared microarray gene expression profiles of A549, HCC827, and NCI-H358 cells before and after EMT. Using an absolute fold-change >5 and a FDR-adjusted *p* value <0.00001 as the cutoff, we observed approximately 30 metabolism-associated genes that consistently demonstrated differential expression in all three mesenchymal and parental cell line comparisons (Additional file 4: Figure S2A). Among them, *PDK4*, but not the other related PDKs (*PDK1-3*), was consistently and dramatically downregulated in all three mesenchymal cell line models (Figure 2B).

The pyruvate dehydrogenase (PDH) complex is a mega-protein complex that functions at the interface of glycolysis and the TCA cycle, critically controlling entry into the TCA cycle by catalyzing the conversion of pyruvate to acetyl-CoA, thereby supplying acetyl-CoA to the TCA cycle [20]. The regulatory mechanisms for PDH activity include its inactivation by four kinases, PDK1-4, and its activation by two phosphatases, PDP1-2 [21]. Decreased expression of PDK4 would therefore be expected to activate PDH and divert glucose to the TCA cycle, consistent with the observed diversion of glucose to TCA cycle/glutamate (Figure 2A).

To validate the microarray findings, we confirmed the reduction in PDK4 expression in TGFβ-treated mesenchymal cells at both the mRNA (Figure 2C) and protein (Figure 2D) levels. Of note, we were only able to reliably detect endogenous PDK4 protein using isolated mitochondria preparations. In addition to lung cancer cell lines, we examined the pancreatic cancer cell line PANC-1 as well as the mammary cell line MCF10A and its oncogenically transformed derivative MCF10AT. All of these cell lines have previously been reported to undergo TGFβ-induced EMT [22, 23] and similarly demonstrated decreased *PDK4* expression following TGFβ treatment (Additional files 4: Figure S2B, C). We also analyzed the time-course of TGFβ treatment and observed decreased *PDK4* mRNA levels as early as 30′ after TGFβ treatment (Additional files 4: Figure S2D, E), suggesting that *PDK4* is regulated by TGFβ signaling at the mRNA level and that *PDK4* downregulation is likely an early event in the induction of EMT by TGFβ.

We next examined whether the decreased expression of PDK4 may be relevant in other models in which EMT was not directly induced by TGFβ. A549 cells that had been rendered resistant to a combination of MEK and PI3K inhibitors (GDC-0973 and GDC-0941, respectively; Shiuh-Ming Luoh and Marcia Belvin, unpublished data) displayed hallmark features of EMT, including increased expression of Vimentin and N-cadherin as well as decreased expression of E-cadherin (Figure 2E). In this model, we similarly observed dramatically reduced PDK4 expression in the resistant, mesenchymal derivatives (Figure 2E).

Finally, we re-introduced PDK4 into the mesenchymal derivative of the HCC827 cells and found that PDK4 overexpression completely reversed glucose diversion to the TCA cycle/glutamate (Figure 2F), indicating that PDK4 loss is indeed responsible for the diversion of glucose to the TCA cycle. Consistent with PDK4’s functional role in such metabolic rewiring, we also anticipated and observed increased PDH activity in the mesenchymal cells as shown by the increased contribution of pyruvate to the TCA cycle/glutamate (Figure 2G). This diversion of pyruvate to the TCA cycle/glutamate was similarly rescued following PDK4 reintroduction into the mesenchymal derivatives (Figure 2G). In contrast, we did not observe any changes in glucose contribution to pyruvate or lactate in the mesenchymal derivatives of HCC827 cells (Additional file 4: Figure S2F). Taken together, these findings strongly implicate the PDH regulatory component PDK4 in EMT.

### PDK4 expression partially prevents TGFβ-induced EMT

**Figure 3:**
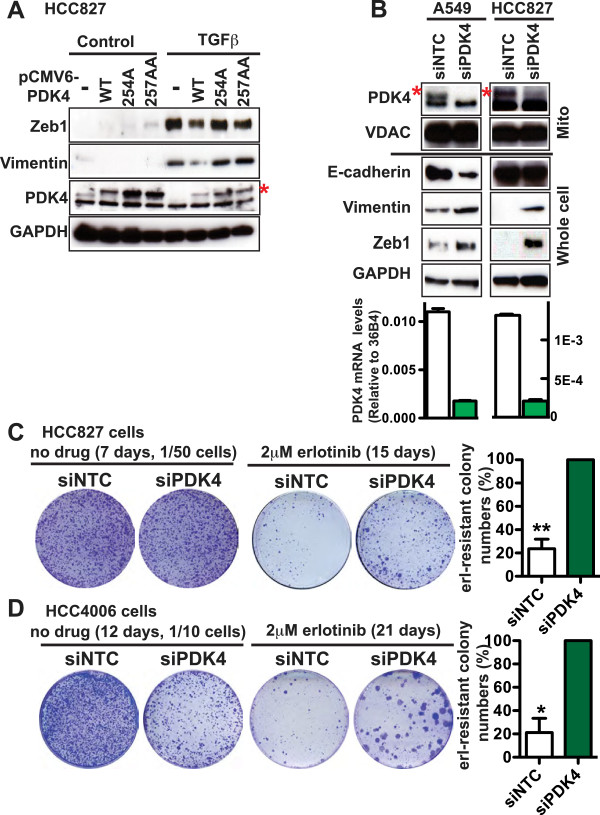
**PDK4 inhibition promotes EMT. (A)** HCC827 cells were transfected with pCMV6-AC-IRES-GFP vector or pCMV6-PDK4-IRES-GFP (WT, 254A, or 257AA), then cultured in the presence of 1.5 μg/ml puromycin for 3 weeks. After that, the GFP-positive cells were enriched through FACS, cultured in the presence or absence of 2 ng/ml TGFβ for 10 days, and lysed for immunoblotting. **(B)** A549 and HCC827 cells were transfected with siNTC pool no. 2 or siPDK4 pool at 1 day and 3 days post-seeding. Two days after the second transfection, the cells were lysed for immunoblotting and qRT-PCR. VDAC and GAPDH are protein loading controls for mitochondrial lysate and whole cell lysate, respectively. In **A** and **B**, the *red asterisk* denotes the upper band that is specific for PDK4. **(C, D)** HCC827 **(C)** and HCC4006 **(D)** cells were transfected with siNTC pool no. 2 or siPDK4 pool at 20 nM siRNA at 1 day and 3 days post-seeding. Twenty-four hours after the second transfection, the media was replaced with growth media containing 2 μM erlotinib and cells were continuously cultured in such media with fresh media replenished every 3 to 4 days for 2 to 3 weeks. For the control plates without erlotinib treatment, the day before the second transfection, a fraction of the cells was re-plated at low density and subjected to a second transfection the next day. At the end of the experiment, cells were stained with crystal violet. The quantification of colony numbers represents four independent experiments, and for each experiment, the colony number in the siNTC plate is normalized to that in the siPDK4 plate. Paired *t*-test was performed for **C** and **D**. Data are plotted as mean ± SEM. *p < 0.05; **p < 0.01.

To determine whether PDK4 plays a functional role during EMT, we tested whether ectopic PDK4 expression would impede EMT. We generated HCC827 cells stably expressing wild-type or kinase-dead PDK4 (Figure 3A). Based on the structure of PDK4 [24], we generated two kinase-dead mutants by mutating the canonical ATP-binding sites—Glu254Ala, referred to as 254A, and Lys257Ala/Asp258Ala, referred to as 257AA. Although we still observed EMT following TGFβ treatment in cells expressing recombinant PDK4, importantly, wild-type PDK4 was indeed able to partially block the expression of the mesenchymal markers Zeb1 and Vimentin, whereas the kinase-dead mutants of PDK4 could not impede EMT. These findings suggest that the kinase activity of PDK4 is required for its role in EMT.

### Inhibiting PDK4 drives an EMT associated with erlotinib resistance

Next, we asked whether blocking PDK4 was sufficient to drive EMT. We specifically knocked down PDK4 using a siRNA smart pool. As shown in Figure 3B, we achieved efficient PDK4 knockdown in A549 and HCC827 cells at both the mRNA and protein level. Notably, in A549 cells, PDK4 knockdown decreased the expression of E-cadherin and modestly increased the expression of Vimentin and Zeb1. In HCC827 cells, PDK4 knockdown markedly increased the expression of Vimentin and Zeb1, although it had no effect on E-cadherin (Figure 3B). To rule out potential off-target effects of the siRNA pool, we performed a deconvolution analysis and found that at least three single siRNAs targeting PDK4 efficiently decreased *PDK4* mRNA levels (Additional file 5: Figure S3A) and E-cadherin in A549 cells (Additional file 5: Figure S3B). These same single siRNAs also increased Vimentin and Zeb1 in HCC827 cells (Additional file 5: Figure S3C, H). To determine whether downregulation of other PDKs could also affect EMT, we individually knocked down PDK1-PDK3 using their respective siRNA smart pool. Under conditions of comparable knockdown efficiency (Additional file 5: Figure S3D), while knockdown of these other PDKs led to a modest effect on the expression of EMT markers, PDK4 knockdown clearly caused the most pronounced effect on the expression of classical EMT markers in A549 (Additional file 5: Figure S3E) and HCC827 (Additional file 5: Figure S3F) cells.

Accumulating evidence has linked EMT to drug resistance [2–6]. Consistent with these findings, HCC827 cells that have been experimentally induced to undergo EMT demonstrate erlotinib resistance (Figure 1C), and conversely, HCC827 cells that have been selected for erlotinib resistance through chronic drug exposure gain mesenchymal features [25]. To address a potential role for PDK4 in EMT-associated erlotinib resistance, we performed PDK4 RNAi studies and examined colony formation capacity in the presence of erlotinib in mutant EGFR-driven HCC827 cells. In the absence of erlotinib, PDK4 knockdown had no inhibitory effect on cell growth (Figure 3C). In contrast, PDK4 knockdown significantly promoted colony formation in the presence of erlotinib (Figure 3C and Additional file 5: Figure S3G), suggesting that its downregulation enhanced erlotinib resistance. This effect was also most pronounced with PDK4 (Additional file 5: Figure S3I) compared to other PDKs. Notably, PDK4 knockdown also promoted erlotinib resistance in *EGFR*-mutant HCC4006 cells (Figure 3D and Additional file 5: Figure S3J), although it inhibited their growth in the absence of erlotinib (Figure 3D). In addition to drug resistance, increased migration and/or invasion are hallmarks of EMT. We examined the migratory and invasive capacity of these cells using scratch wound and boyden chamber assays, respectively, and observed increased migration (Additional file 6: Figure S4A) and invasion (Additional file 6: Figure S4B) in A549 cells following PDK4 knockdown. Collectively, these data strongly suggest that PDK4 inhibition can promote erlotinib resistance and EMT.

Considering PDK4’s role as a metabolic inhibitor of PDH that controls entry into the TCA cycle, we examined the effect of siPDK4 on metabolism using the MIDA and Seahorse assays. We observed an increased contribution to glutamate from glucose (Additional file 7: Figure S5A) as well as a modestly decreased glycolysis/OXPHOS ratio (Additional file 7: Figure S5B) in siPDK4 cells, indicating that PDK4 knockdown promotes a metabolic reprogramming event that favors the TCA cycle. Considering that OXPHOS is more efficient in glucose utilization, we hypothesized that siPDK4 would enhance cell survival under low-glucose conditions. Indeed, in A549 cells in which PDK4 had been knocked down, we observed a robust increase in cell survival under low-glucose conditions (Additional file 7: Figure S5C), but only modestly enhanced survival under low-glutamine conditions (Additional file 7: Figure S5D).

### AIF interacts with PDK4 and promotes EMT, erlotinib resistance, and ROS production

**Figure 4:**
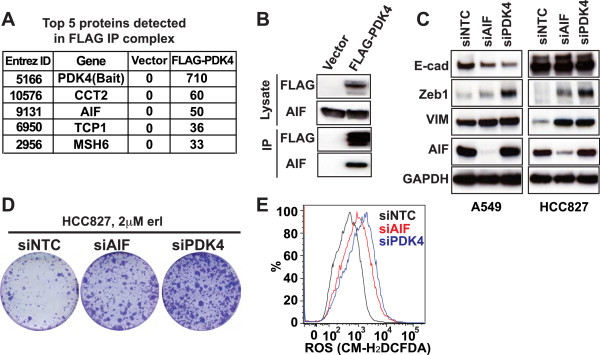
**AIF interacts with PDK4 and plays a role in EMT. (A)** HEK293T cells were transfected with the pCMV6-Entry empty vector or pCMV6-FLAG-Myc-PDK4. Forty-eight hours post-transfection, the cells were lysed and FLAG IP was performed. Mass spectrometry was performed to analyze the immunoprecipitates, and the top five proteins identified in the immunoprecipitates are shown. **(B)** Immunoblotting was performed with samples prepared as in **A** to confirm the PDK4-AIF interaction. **(C)** A549 cells and HCC827 cells were transfected with siNTC pool no. 2, siAIF pool, or siPDK4 pool at 1 day and 3 days post-seeding, then lysed for immunoblotting. **(D)** HCC827 cells were transfected with siNTC pool no. 2, siAIF pool, and siPDK4 pool, treated with erlotinib (*erl*), and stained with crystal violet as in Figure 3C. **(E)** HCC827 cells were transfected as in **D**. Two days after the second transfection, the cells were stained with CM-H_2_DCFDA and ROS levels were analyzed using flow cytometry.

To gain additional insight into PDK4’s role in EMT, we sought to identify proteins that interact with PDK4. Considering the very limited information regarding PDK4-interacting proteins, we performed an unbiased immunoprecipitation (IP) mass spectrometry experiment to identify novel PDK4-binding proteins. We transiently overexpressed FLAG-tagged PDK4 in HEK293T cells and examined the proteins that specifically bound to PDK4 using IP, followed by mass spectrometry (Additional file 8: Table S2). Interestingly, AIF was one of the proteins that showed a prominent interaction with PDK4 (Figure 4A and Additional file 8: Table S2), which we confirmed by IP-Western blot analysis (Figure 4B).

Consistent with PDK4’s role in mitochondria, AIF is a mitochondrial protein that can translocate to the nucleus and promote caspase-independent apoptosis [26]. Biochemical analysis of AIF has revealed two major functional domains: 1) a DNA binding domain that promotes chromatin condensation and DNA fragmentation and 2) an oxidoreductase domain that is involved in cellular redox metabolism and mitochondrial bioenergetics [26]. Of note, AIF inhibition has also been shown to promote drug resistance to the multi-kinase inhibitor sorafenib and the IκB kinase inhibitor BMS-345541 in melanoma [27, 28].

To establish a potential role for AIF in EMT, we knocked down AIF using a siRNA smart pool and observed EMT in both A549 and HCC827 cells. Similar to PDK4, knockdown of AIF resulted in increased Zeb1 and Vimentin (A549 and HCC827 cells) and decreased E-cadherin (A549 cells) (Figure 4C) and promoted erlotinib resistance (Figure 4D), all of which are consistent with both AIF and PDK4 having a role in EMT. To rule out a potential off-target effect of siAIF on EMT, we deconvoluted the siRNA smart pool in A549 cells and observed that each of the four individual siRNA oligos decreased E-cadherin expression (Additional file 9: Figure S6). Considering AIF’s role in redox regulation, we also measured reactive oxygen species (ROS) levels and detected increased ROS following knockdown of either PDK4 or AIF (Figure 4E). These collective data support a functional requirement for the interaction between AIF and PDK4 in EMT and associated drug resistance.

### *PDK4*-low expression predicts poor prognosis in lung cancer and is frequently down-regulated in human cancer

**Figure 5:**
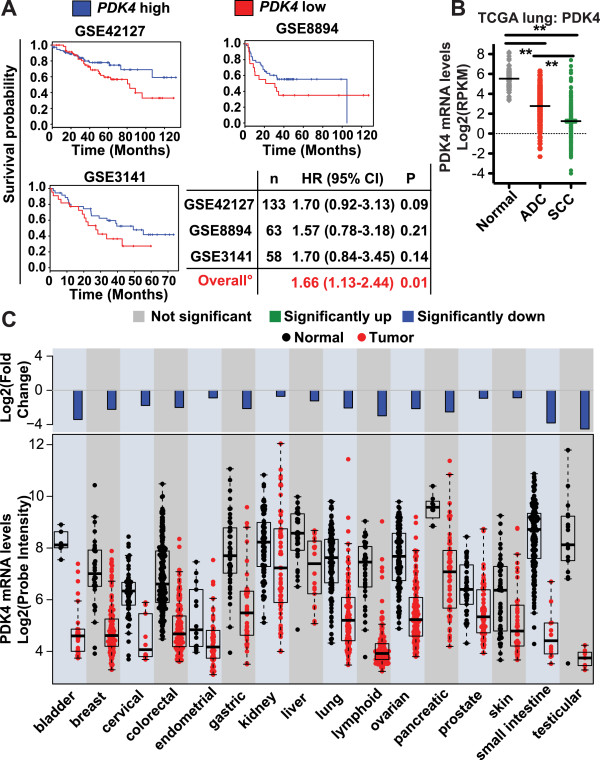
***PDK4*** **-low is associated with poor prognosis in human NSCLC, and PDK4 is frequently downregulated in human cancer. (A)** Kaplan-Meier survival analysis of three independent lung adenocarcinoma patient cohorts shows that *PDK4*-low expression is associated with poor prognosis. Patients were stratified into *PDK4*-high (*blue line*, *PDK4* expression above median) and *PDK4*-low (*red line*, *PDK4* expression below median). Cox hazard ratios of both the individual studies and the meta-analysis of all three studies together are shown. **(B)** *PDK4* mRNA levels in normal tissue, adenocarcinoma (ADC) and squamous cell carcinoma (SCC) of the lung. The data are from RNA-seq analysis of the TCGA NSCLC samples. Data are plotted as mean ± SEM. **p < 0.01. **(C)** *PDK4* mRNA levels in multiple cancer types and their corresponding normal tissues from the Gene Logic database. The fold-change between the mean tumor and the mean normal tissue expression is shown in the bar plot on a log_2_-transformed scale. Significance was determined using a two-tailed *t*-test. Expression levels are shown in the boxplot on a log_2_-transformed scale, with display of the median expression level. The *box* represents 25–75 % percentile. For all indications shown in **C**, *p* < 0.05.

EMT in cancer cells is associated with increased metastasis and resistance to therapy, leading to poor clinical prognosis [2]. To determine whether *PDK4* expression is associated with prognosis, we examined *PDK4* mRNA expression in NSCLC clinical samples. We divided lung adenocarcinoma patient samples into *PDK4*-high (*PDK4* expression above median) and *PDK4*-low (*PDK4* expression below median). We did not observe any association between tumor stages and *PDK4* expression levels. However, in three independent studies (GSE42127, GSE8894, and GSE3141) [29–31], we observed a significant (*p* = 0.01) overall association between *PDK4*-low tumors and poor prognosis. Thus, lung adenocarcinoma patients whose tumors have low *PDK4* expression showed reduced overall survival (Figure 5A).

We further examined *PDK4* expression levels in NSCLC biopsies from the Cancer Genome Atlas (TCGA) using RNA-seq data. We observed dramatically decreased *PDK4* in lung cancer biopsies compared to the corresponding normal tissue (Figure 5B). Of the two different NSCLC subtypes, adenocarcinoma (ADC) and squamous cell carcinoma (SCC), we found that *PDK4* expression was particularly low in SCC, a subtype lacking good treatment options.

To explore *PDK4* expression levels in a broad range of cancer types, we surveyed Gene Logic microarray data covering multiple types of human tumor biopsies and normal tissues. Most notably, we observed dramatically decreased *PDK4* expression in the majority of cancer types examined, including breast, colorectal, lung, lymphoid, ovary, and skin cancers (Figure 5C). Finally, we analyzed the global gene expression changes of 19 cancer types compared to corresponding normal tissue in the Gene Logic database. We ranked the approximately 19,000 genes according to the average fold-change and found that *PDK4* was one of the genes that showed the most dramatic expression loss in cancer (ranked no. 28 overall, data not shown).

## Discussion

Cancer cells preferably use aerobic glycolysis to generate energy, which has been recognized as a hallmark of cancer. Our findings reveal a metabolic rewiring event that drives cancer cells back to an OXPHOS state during the process of EMT or during the acquisition of drug resistance. This observation is consistent with three recent studies: Firstly, Haq *et al.* reported that BRAF inhibitor-resistant cells are more addicted to OXPHOS [32]; secondly, Roesch *et al.* demonstrated that the multi-drug resistant JARID1B^high^ subpopulation of melanoma cells expressed more OXPHOS enzymes [33]; and most recently, Viale *et al.* showed that pancreatic tumor cells surviving oncogene ablation depend on mitochondria [34]. In this study, we analyzed metabolic activity, metabolite profiles, and mass isotopologue distribution to reveal that cancer cells that have undergone EMT divert more glucose to the TCA cycle compared to their parental epithelial cells, which presumably enables the mesenchymal cells to use the metabolites of the TCA cycle as the backbone to produce more amino acids. We further speculate that the increased supply of macromolecules provides the building blocks for *de novo* protein synthesis and extracellular matrix remodeling, which is essential for EMT [35]. Additionally, since OXPHOS is a more efficient process for energy production, shifting to OXPHOS might enhance the ability of cancer cells to survive under conditions of stress, such as drug treatment.

Our study reveals PDK4 as a novel metabolic regulator of EMT and drug resistance. A previous study comparing basal and luminal subtypes of breast cancer demonstrated loss of the metabolic enzyme FBP1 in the more mesenchymal, basal subtype; however, inhibition of FBP1 alone was not sufficient to regulate EMT [36]. Unlike FBP1, we show that inhibition of PDK4 alone is sufficient to induce EMT, and ectopic expression of PDK4 could partially prevent TGFβ-induced EMT, although PDK4 is not differentially expressed between the basal and luminal subtypes of breast cancer (data not shown). As a phenotypic readout of EMT, drug resistance is also enhanced by PDK4 knockdown. In the absence of drug, PDK4 inhibition does not promote, and even inhibits, cell growth in some cell lines; in the presence of erlotinib, PDK4 inhibition dramatically promotes colony formation. This functional profile is reminiscent of a recent study examining MED12, a component of the transcriptional MEDIATOR complex that regulates TGFβ receptor II [13]. Similar to PDK4 inhibition, MED12 inhibition impeded cell growth in the absence of drug, but promoted colony formation in the presence of drug. Whereas MED12 regulates TGFβ signaling, PDK4 appears to be regulated by TGFβ signaling.

Four isoforms of PDK (PDK1-PDK4) with similar structure negatively regulate the activity of PDH. PDK4 may have some unique functions and may also be subject to distinct regulatory mechanisms from those affect PDK1-3. *PDK4* expression has been shown to be regulated by various metabolic stimuli (such as starvation, exercise, and diabetes), the transcription factors FOXO and E2F1 [20], and epigenetic mechanisms such as promoter methylation and histone acetylation [37, 38]. Although PDK1 and PDK2 have been previously proposed as cancer drug targets [39, 40], our findings suggest that PDK4 may function as a metabolic tumor suppressor. We observed that depletion of PDK4 by RNAi promoted colony formation in EGFR-mutant cell lines upon EGFR inhibition. Conversely, Grassian *et al.* reported that PDK4 overexpression suppressed the proliferation of normal mammary epithelial MCF10A cells [41]. In that study, increased PDK4 levels and decreased flux through PDH was induced upon extracellular matrix (ECM) detachment, leading to the metabolic impairment of these cells. The fact that PDK4 emerged as a key factor during EMT in our study further establishes the role of this enzyme in regulating ECM/tumor crosstalk and bioenergetics. Significantly, we observed widespread loss of PDK4 expression in tumor cells compared to normal tissue. This loss of *PDK4* expression in cancer is more substantial and prevalent than that of most known tumor suppressors, and collectively, these findings suggest that PDK4 may function as a metabolic tumor suppressor, a possibility that would need to be further explored in an *in vivo* tumorigenesis study.

The mechanism(s) by which PDK4 regulates EMT and drug resistance is not completely clear. We speculate that PDK4 depletion plays a role in the survival of drug-tolerant persisters, but may not be essential for the proliferative expansion of these cells. Furthermore, since PDK4 knockdown induces Zeb1 expression, PDK4 likely functions upstream of the EMT-associated transcription factors. Our data also suggest a novel interaction between AIF and PDK4, both physically and functionally. We show the knockdown of either AIF or PDK4 promoted EMT, erlotinib resistance, and ROS production. Indeed, AIF inhibition has been previously shown to promote resistance to serum deprivation-induced cell death in embryonic stem cells [42] as well as to a multi-kinase inhibitor and an IκB kinase inhibitor in melanoma [27, 28]. A hypothesis that remains to be tested is that PDK4 or AIF inhibition promotes EMT and drug resistance through ROS generation, especially given the established role of ROS in physiological conditions [43, 44].

## Conclusions

Our collective observations implicate a glycolysis-to-OXPHOS shift in drug-resistant cancer cells that have undergone EMT. We provide evidence that downregulation of PDK4 is responsible for such metabolic rewiring, is sufficient to drive EMT, and promotes erlotinib resistance in *EGFR* mutant lung cancer cells. In addition, we have identified a novel PDK4-interacting protein, AIF, which plays a role in EMT and drug resistance. Finally, analysis of human lung adenoma tumor samples reveals *PDK4*-low as a predictor of poor prognosis, consistent with PDK4’s role in EMT. Although establishing the precise mechanism by which PDK4 regulates EMT will require further investigation, the findings described here implicate a specific metabolic reprogramming event in EMT associated with resistance to cancer treatments, thereby revealing novel potential therapeutic opportunities.

## Electronic supplementary material

Additional file 1: **Extended Methods.** (DOC 52 KB)

Additional file 2: Figure S1: Metabolic rewiring is observed during EMT. **(A)** *EGFR*-mutant HCC4006 cells were cultured in the presence of erlotinib (erl) for 3 months to establish erlotinib resistance. The Glycolysis/OXPHOS ratio of the HCC4006 parental and erl-resistant cells (plotted as PPR/OCR), as measured by the Seahorse metabolic analyzer. Technical replicates from six wells of a 96-well plate are shown, and the experiment was repeated twice. **, *p* < 0.01. **(B, C)** Summary from global metabolomic profiling of cells treated as in Figure 1. See Table S1 for the complete metabolomics data. **(B)** The ten metabolites that changed consistently upon EMT across the three tested cell line models. **(C)** Changes in cellular amino acid levels after EMT in three tested cell line models. **(D)** The growth rates of the parental cells and their corresponding mesenchymal derivatives. Cell density (%) was recorded by IncuCyte every 4 hours. **(E)** Parental (P) cells and corresponding mesenchymal (M) derivatives were incubated with growth media containing ^13^C-U-glutamine overnight, and then subjected to LC-MS. Glutamine to glutamate contribution was plotted based on the percentage of (M + 5) glutamate in the total glutamate pool. Each data point is from three to five separate biological samples generated at the same time. Data are plotted as mean +/-SEM. **, *p* < 0.01. (PDF 174 KB)

Additional file 3: Table S1: Global metabolomic profiling of the parental and mesenchymal derivatives of A549, HCC827 and NCI-H358 cells. (XLS 455 KB)

Additional file 4: Figure S2: PDK4 is down-regulated during EMT. **(A)** Differentially expressed metabolism-associated genes before and after EMT in three NSCLC lines based on the microarray analysis. **(B)** *PDK4* mRNA levels in pancreatic cancer cell line PANC1 before (Parental, P) and after (Mesenchymal, M) TGFβ-induced EMT. **(C)** *PDK4* mRNA levels in normal breast epithelial cell line MCF10A and its tumorigenic derivative MCF10AT cells before (Parental, P) and after (Mesenchymal, M) TGFβ-induced EMT. **(D, E)** Time course of *PDK4* mRNA levels in response to TGFβ treatment in A549 cells **(D)** and HCC827 cells **(E)**. *PDK4* mRNA levels were quantified by qRT-PCR. **(F)** Cells were treated and analyzed as in Figure 2F. Glucose to pyruvate or lactate contribution was plotted based on the percentage of (M + 3) pyruvate or (M + 3) lactate in the total pyruvate or lactate pool, respectively. (PDF 134 KB)

Additional file 5: Figure S3: Deconvolution of the siPDK4 smart pool, and the effects of PDK1-PDK3 knockdown on EMT. **(A-C)** A549 and HCC827 cells were transfected with siPDK4 pool or three individual siRNAs from the pool at one day and three days post-seeding. Two days after the second transfection, the cells were lysed for immunoblotting and qRT-PCR. **(A)** *PDK4* knockdown (k/d) efficiency from individual siRNAs in A549 cells, evaluated using qRT-PCR. **(B)** Immunoblots showing the effects of PDK4 knockdown on the epithelial marker E-cadherin in A549 cells, using three individual siRNAs. **(C)** Immunoblots showing the effects of PDK4 knockdown on mesenchymal markers Vimentin and Zeb1 in HCC827 cells, using three individual siRNAs. **(D-F)** A549 and HCC827 cells were transfected with siRNA smart pools of siNTC, siPDK1, siPDK2, siPDK3 or siPDK4 at one day and three days post-seeding. **(D)** Validation of knockdown (k/d) efficiency of each PDK siRNA on the corresponding *PDK* isoform, quantified by qRT-PCR. The y-axis represents the particular *PDK* mRNA levels in siPDK-transfected cells over siNTC-transfected cells. **(E)** Immunoblots showing the effects of each PDK isoform knockdown on the epithelial marker E-cadherin in A549 cells. **(F)** Immunoblots showing the effects of each individual PDK isoform knockdown on the mesenchymal markers Vimentin and Zeb1 in HCC827 cells. **(G)** Colony formation capacity of HCC827 cells treated as in **C**, in the presence of 2 μM erlotinib. **(H)** *PDK4* knockdown (k/d) efficiency using individual siRNAs in HCC827 cells, as evaluated in **A**. **(I)** Colony formation capacity of HCC827 cells treated in **F**, in the presence of 2 μM erlotinib. The siNTC and siPDK4 plates in **I** are reproduced from Figure 3C to facilitate a direct comparison amongst all parameters. **(J)** Colony formation capacity of HCC4006 cells treated as in **G**, in the presence of 2 μM erlotinib. (PDF 210 KB)

Additional file 6: Figure S4: PDK4 knockdown promotes cell migration and invasion. A549 cells were transfected with siNTC pool#2 or the siPDK4 pool at one day and three days post-seeding. The day after the second transfection, cells were seeded in an IncuCyte ImageLock plate for migration assay **(A)**, and a Boyden chamber for invasion assay **(B)**, as described in the Extended Methods. The migration assay shows the average of 10 wells from one experiment, which is representative of two independent experiments. The invasion assay is the average of two independent experiments each containing two replicates. *, *p* < 0.05. (PDF 262 KB)

Additional file 7: Figure S5: PDK4 knockdown promotes metabolic rewiring and cell survival under low glucose conditions. HCC827 cells were transfected with siNTC pool#2 or the siPDK4 pool at one day and three days post-seeding. **(A)** The day after the second transfection, the cells were incubated overnight with growth media containing ^13^C-U-glucose, and then subjected to LC-MS. Glucose to glutamate contribution was plotted based on the percentage of (M + 2) glutamate in the total glutamate pool. Each data point is from three separate biological samples generated at the same time. **(B)** The day after the second transfection, the cells were seeded in Seahorse plates to measure Glycolysis/OXPHOS ratio (defined by PPR/OCR). Each data point is from eight wells. **(C, D)** The day after the second transfection, cells were plated in 96 well plates. After overnight incubation, cells were switched to media containing various concentrations of glucose **(C)** or glutamine **(D)**. Three days after, cell survival was measured using CellTiterGlo. Each data point is an average of three wells. Data are plotted as mean +/-SEM. *, *p* < 0.05, **, *p* < 0.01. (PDF 311 KB)

Additional file 8: Table S2: Proteins identified in the FLAG-PDK4 immunoprecipitates by mass spectrometry analysis. (XLS 104 KB)

Additional file 9: Figure S6: Deconvolution of the siAIF smart pool. A549 cells were transfected with siNTC pool#2, the siAIF smart pool, or individual siRNAs in the siAIF smart pool, at one day and three days post-seeding, then lysed for immunoblotting. (PDF 437 KB)
