# Supplementary material for: Metabolic and transcriptional profiling reveals pyruvate dehydrogenase kinase 4 as a mediator of epithelial-mesenchymal transition and drug resistance in tumor cells
Source: Cancer Metab. 2014 Nov 3;2:20. doi: 10.1186/2049-3002-2-20 (PMC4221711; doi:10.1186/2049-3002-2-20)
Supplement: Supplementary file 1 — Additional file 1: Extended Methods. (DOC 52 KB) [file 40170_2014_136_MOESM1_ESM.doc]

**Extended Methods**

**Amino acid analysis from culture media**

Cells in a volume of 100 μl were plated in six replicates in 96-well tissue culture plates to reach ~80% confluence the next day. The next day, the spent media was harvested, spun down, and the supernatants were collected. The cells on the plate were fixed with 4% paraformaldehyde containing Hoechst33342 (Pierce) for 15 minutes, and then the total cell number (nuclei number) per well was counted using a semi-automated ImageExpress high throughput microscope (Molecular Devices). The glutamate concentration in the media was measured using the YSI2700 biochemistry analyzer (YSI life sciences), following manufacture’s protocol.

**Cell invasion assay**

Pre-labeled DilC12(3) (BD Biosciences) cell suspensions in serum-free RPMI media were added to the apical chamber of the BD BioCoat™ Tumor Invasion System, 8µm, at 3 x 10^5^ cells per chamber. RPMI containing 10% FBS was added as the chemoattractant to the bottom chamber. Chambers were incubated for 18hr at 37^o^C, 5% CO_2_, and invasion of fluorescent cells was determined at a wavelength of 549/565 nm (Ex/Em) using SpectraMax® M5 (Molecular Devices).

**Cell migration assay**

20,000 cells were cultured in 96-well IncuCyte ImageLock plates. After 24 hours, the Essen 96-well WoundMaker was used to generate consistent wounds in the confluent monolayer. Cell migration was monitored by the IncuCyte imaging system for an additional 24 hours. The data was analyzed by the IncuCyte software package.

**Co-immunoprecipitation and protein mass spectrometry**

293T cells were transiently transfected with pCMV6-Entry-PDK4 plasmid or empty vector (Origene). 72 hours after transfection, cells were scraped and pelleted, lysed in lysis buffer (20 mM Tris pH 7.8, 3 mM MgCl_2_, 100 mM NaCl, 0.1% Triton-X100). The cleared lysate was incubated with anti-FLAG M2 sepharose beads (Sigma) for 2 hours at 4^0^C, then washed twice with lysis buffer, once with high salt buffer (20 mM Hepes pH 7.9, 1.5 mM MgCl_2_, 420 mM NaCl, 0.2 mM EDTA, 25% glycerol) and 5 times with low salt buffer (20 mM Tris pH 7.4, 300 mM NaCl, 0.2 mM EDTA, 20% glycerol, 0.1% NP-40). The final low salt wash was incubated at 4^0^C overnight with agitation. The next day, the beads were washed in TBS, then resuspended in 1x SDS sample buffer, and subjected to either western blotting or mass spectrometry . For mass spec analysis, cells from four 500 cm^2^ plates were used to prepare each sample. For western blotting, cells from one 15-cm plate were used to prepare each sample.

**Drug sensitivity assays**

Drug sensitivity was measured either by a standard 3-day CellTiter-Glo viability assay (Promega) as previously described or a long-term colony formation assay, in the presence of erlotinib (LC labs). For the latter, cells at >50% confluence were cultured in the presence of 2 μM erlotinib for two to three weeks, with fresh media containing drug replenished every three days, and colonies were counted manually assisted with the Cell Counter plug-in of Image J.

**Mass spectrometry analysis of metabolites**

Glutathione, glutamine, and glutamate isotopologues were measured by liquid chromatography – electrospray ionization tandem mass spectrometry on a hybrid triple quadrupole ion trap mass spectrometer (4000 QTRAP, AB Sciex). Frozen cell pellets were thawed on ice and quenched by addition of -20^0^C methanol in approximately equal volume to the pellet in order to achieve a 50% final methanol concentration by volume. The resulting suspensions were immediately lysed by four 1-second pulses of an ultrasonic cell disruptor (Microson). Cell debris was pelleted by 14,000 RCF centrifugation for 10 minutes at 4⁰C. The supernatants were transferred to 1.5mL centrifuge tubes (Eppendorf), dried by centrifugation under vacuum and stored at -20^0^C until use. After that, the dried samples were re-dissolved in 0.1% trifluoroacetic acid (TFA), centrifuged at 14,000 RCF for 5 minutes, and used as is or diluted up to 50-fold with 0.1% TFA depending on the volume of the cell pellet. DTT was added to the samples to convert oxidized glutathione (GSSG) to the reduced form (GSH). Two microliters of each sample were injected onto a 2mm x 150mm C18 column (MetaSil AQ 3μm; Agilent) and eluted isocratically at 2% acetonitrile in water with 0.1% formic acid by volume at a flow rate of 75 μL/minute. The column was washed and regenerated with a 2.5 minute gradient to 60% acetonitrile, 0.1% formic acid at 200μL per minute, held at that solvent composition for 30 seconds, returned to 2% acetonitrile, 0.1% formic acid and equilibrated for 10 minutes before slowing to 75 μL per minute for the next injection.

Three scan functions were repeated in a cycle throughout the chromatographic elution in order to selectively detect all isotopologues of glutamine, glutamate, and glutathione. These were, respectively, neutral loss scans for 17.0 and 18.0 Da, and a precursor ion scan for parents of m/z 77.0. The neutral loss scans were each acquired over a parent ion range from m/z 145 to 155 in 0.25 seconds, with quadrupoles 1 and three operated at unit resolution. The precursor ion scan covered m/z 306 to 319 in 0.5 seconds. Quadrupole 1 was operated at unit resolution whereas quadrupole 3 was operated with “open” resolution so as to pass the y1 ion of glutathione in each of its possible isotopologues (m/z 76.0, 77.0, 78.0). Retention times for each analyte were determined from purified standards, and were approximately 4.6, 4.8, and 6.9 minutes for glutamine, glutamate, and glutathione respectively. Spectra spanning the 50% maximum of each chromatographic peak in the appropriate scan function at these times were summed, and the peak areas exported.

Citrate, α-ketoglutarate, and malate isotopologues were measured by liquid chromatography –triple quadrupole time-of-flight mass spectrometer (TripleTOF 5600, AB Sciex). Cells were washed once with PBS, lysed and scraped in cold lysis buffer composed of acetonitrile: methanol: H_2_O (4.5: 4.5: 1) on ice, sonicated in a water bath for 30 seconds, then spun at 14,000 RCF at 4^0^C for 10 mins. Supernatants were collected for mass spec analysis. Ten microliters of each sample were injected onto a 3mm x 150mm StableBond column (ZORBAX AQ 5μm; Agilent) and eluted isocratically in water with 0.1% formic acid by volume at a flow rate of 500 μL/minute.  Data collected with TOF MS scan function at 400-msec scan time and 4.5-minute run time.  Monoisotopic m/z with 10 mDa window was used for data integration of each analyte.  Retention time for each analyte was confirmed with purified standards.  MultiQuant™ Software (AB sciex) was used for data analysis.

Isotopologue abundances were not corrected for the natural abundance of ^13^C, which in these experiments was small compared to the contribution of the isotopic tracers introduced in the experiment.

**Mitochondrial isolation**

Mitochondria for western blotting were isolated using a magnetic beads-based mitochondrial isolation kit (Miltenyi Biotech), following vendor’s protocol. Ten million cells were used for each sample. After being eluted from the column, the mitochondria were pelleted and washed using the storage buffer provided in the kit, then resuspended in RIPA lysis buffer for western blotting analysis.

**Plasmids**

The Precision LentiORF vectors pLOC-RFP and pLOC-PDK4 were from Open Biosystems. The pCMV6-Entry-PDK4 plasmid containing FLAG tag was purchased from Origene. The pCMV6-PDK4-IRES-GFP expression plasmids used in Figure 3A were generated by inserting PDK4 wild-type from the pCMV6-Entry vector into pCMV6-AC-IRES-GFP-puro empty vector (Origene), between the BamH I and Not I sites.

**Quantitative RT-PCR**

RNA was isolated using the RNeasy RNA isolation kit (Qiagen). RNA was subjected to cDNA synthesis using the High Capacity Reverse Transcription Kit (Applied Biosystems), followed by real-time PCR using Taqman universal master mix (Applied Biosystems). Alternatively, RNA was directly subjected to real-time PCR using Taqman Real-time PCR one-step master mix (Applied Biosystems). The genes and the Taqman assay IDs (Applied Biosystems) are listed as follows: PDK1 (Hs01561850_m1), PDK2 (Hs00176865_m1), PDK3 (Hs00178440_m1), PDK4 (Hs01037712_m1), and RPLPO 36B4 (4333761F). RPLPO 36B4 was used as the internal control for normalization.

**ROS measurement**

Cells in 6-well plates were incubated with growth media containing 5 μM CM-H_2_DCFDA (Invitrogen) for 30 mins, rinsed with PBS and trypsinized. After being spun down, the cells were resuspended in growth media containing propidium iodine (Invitrogen). Flow cytometry analysis for H_2_DCFDA staining in the live cells was performed immediately. Data were analyzed and plotted in FlowJo.

**RNA interference**

Knockdown of gene expression was achieved by two sequential transfections using siGENOME SMARTpool siRNAs (Dharmacon) and Lipofectamine RNAiMax (Invitrogen). A pool of non-targeting siRNAs (siGENOME SMARTpool control, non-targeting #2) (Dharmacon) was used as the negative control.

**Supplementary References**

1. Huang X, Summers MK, Pham V, Lill JR, Liu J, Lee G, et al. Deubiquitinase USP37 is activated by CDK2 to antagonize APC(CDH1) and promote S phase entry. Molecular cell. 2011;42:511-23.

2. Sun Y, Wang BE, Leong KG, Yue P, Li L, Jhunjhunwala S, et al. Androgen deprivation causes epithelial-mesenchymal transition in the prostate: implications for androgen-deprivation therapy. Cancer Res. 2012;72:527-36.
