# Supplementary figures and images for: Metabolic and transcriptional profiling reveals pyruvate dehydrogenase kinase 4 as a mediator of epithelial-mesenchymal transition and drug resistance in tumor cells
Source: Cancer Metab. 2014 Nov 3;2:20. doi: 10.1186/2049-3002-2-20 (PMC4221711; doi:10.1186/2049-3002-2-20)

# Figure S1

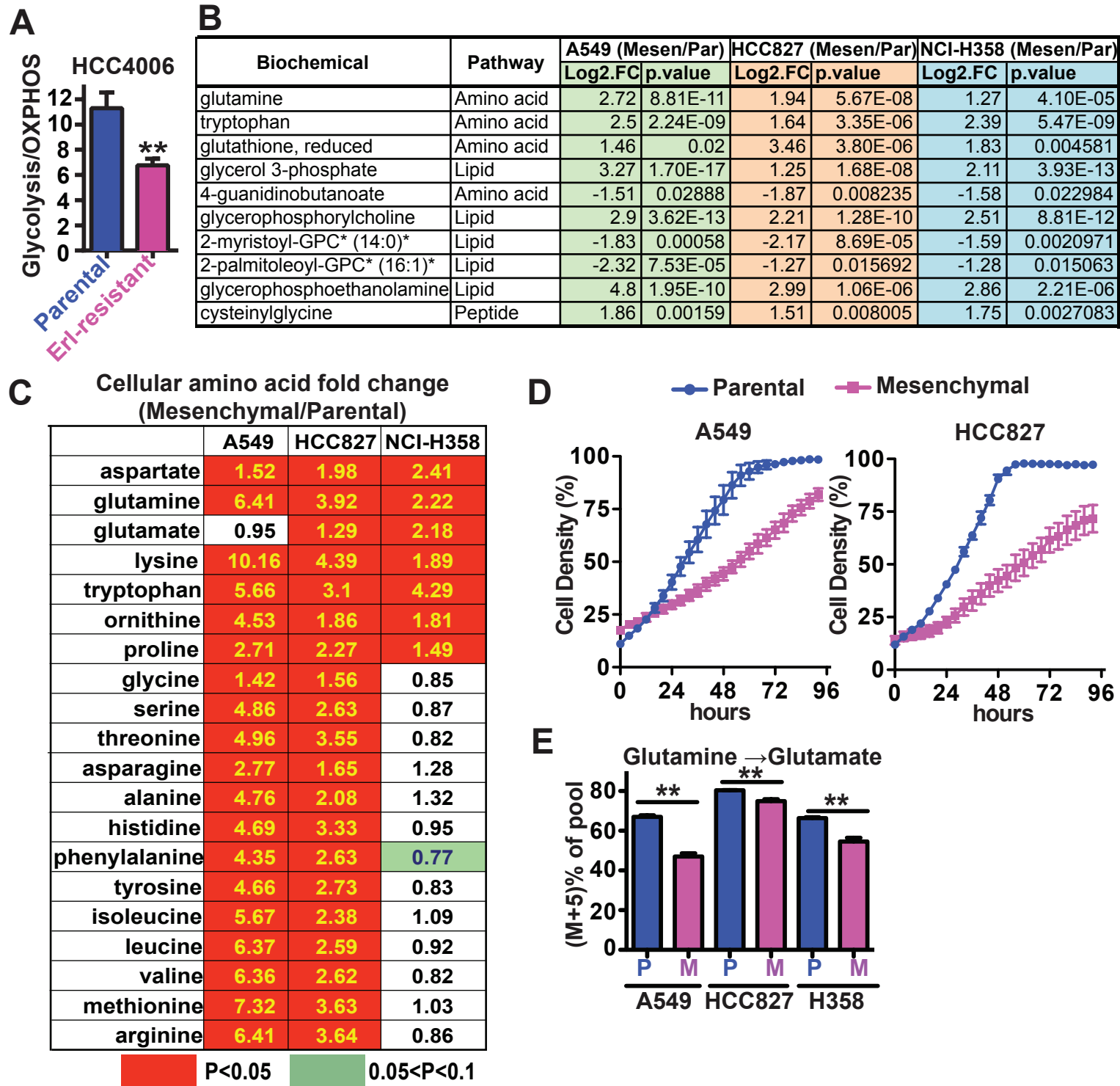

Supplement: Supplementary file 2 — Additional file 2: Figure S1: Metabolic rewiring is observed during EMT. (A) EGFR-mutant HCC4006 cells were cultured in the presence of erlotinib (erl) for 3 months to establish erlotinib resistance. The Glycolysis/OXPHOS ratio of the HCC4006 parental and erl-resistant cells (plotted as PPR/OCR), as measured by the Seahorse metabolic analyzer. Technical replicates from six wells of a 96-well plate are shown, and the experiment was repeated twice. **, p < 0.01. (B, C) Summary from global metabolomic profiling of cells treated as in Figure 1. See Table S1 for the complete metabolomics data. (B) The ten metabolites that changed consistently upon EMT across the three tested cell line models. (C) Changes in cellular amino acid levels after EMT in three tested cell line models. (D) The growth rates of the parental cells and their corresponding mesenchymal derivatives. Cell density (%) was recorded by IncuCyte every 4 hours. (E) Parental (P) cells and corresponding mesenchymal (M) derivatives were incubated with growth media containing 13C-U-glutamine overnight, and then subjected to LC-MS. Glutamine to glutamate contribution was plotted based on the percentage of (M + 5) glutamate in the total glutamate pool. Each data point is from three to five separate biological samples generated at the same time. Data are plotted as mean +/-SEM. **, p < 0.01. (PDF 174 KB) [file 40170_2014_136_MOESM2_ESM.pdf]

# Figure S2

## A

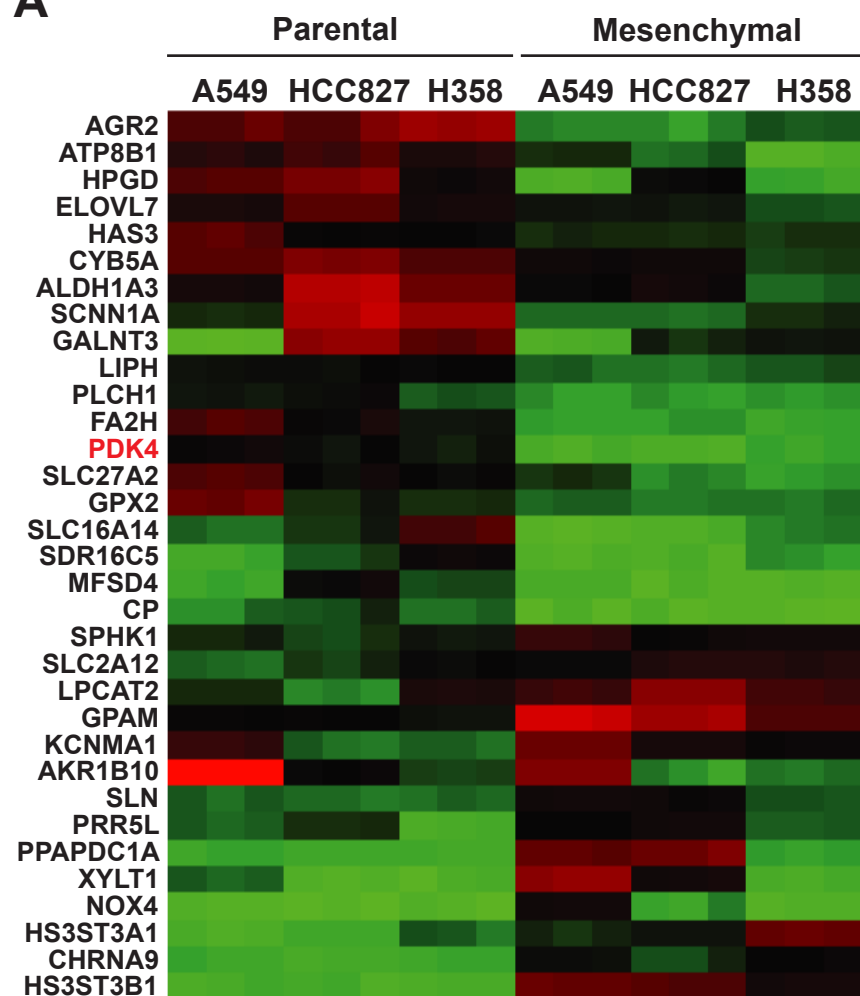

Value

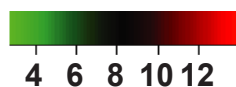

## B

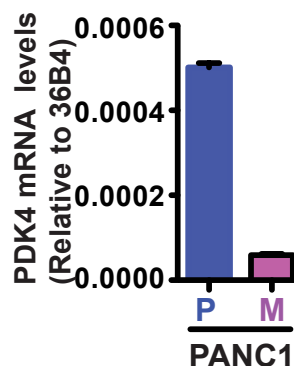

## C

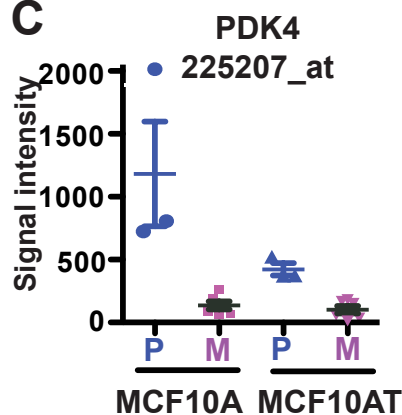

## D

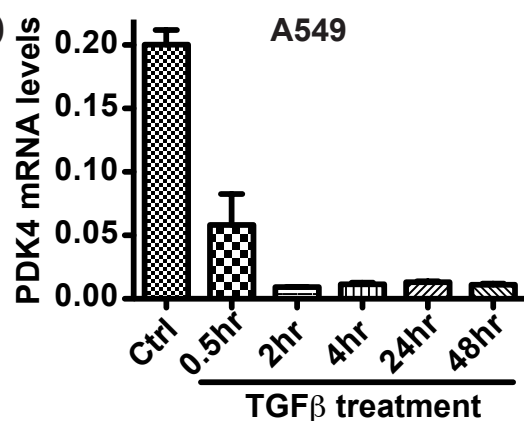

## E

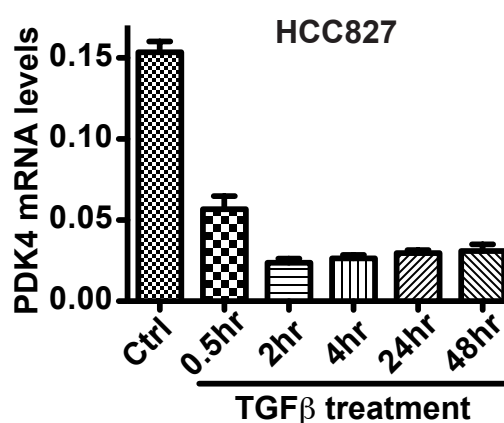

## F

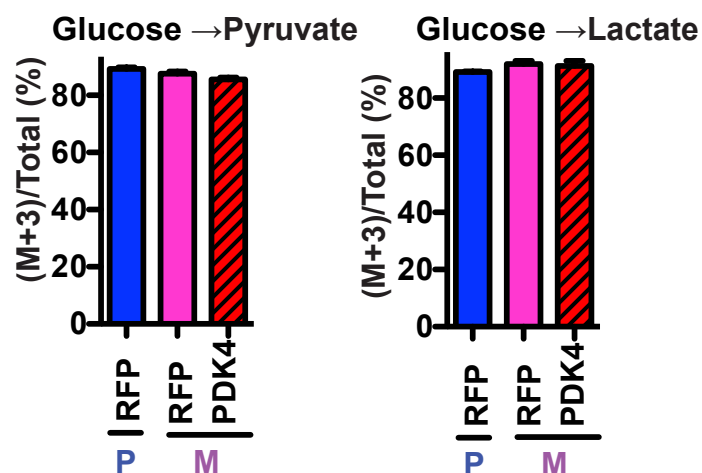

Supplement: Supplementary file 4 — Additional file 4: Figure S2: PDK4 is down-regulated during EMT. (A) Differentially expressed metabolism-associated genes before and after EMT in three NSCLC lines based on the microarray analysis. (B) PDK4 mRNA levels in pancreatic cancer cell line PANC1 before (Parental, P) and after (Mesenchymal, M) TGFβ-induced EMT. (C) PDK4 mRNA levels in normal breast epithelial cell line MCF10A and its tumorigenic derivative MCF10AT cells before (Parental, P) and after (Mesenchymal, M) TGFβ-induced EMT. (D, E) Time course of PDK4 mRNA levels in response to TGFβ treatment in A549 cells (D) and HCC827 cells (E). PDK4 mRNA levels were quantified by qRT-PCR. (F) Cells were treated and analyzed as in Figure 2F. Glucose to pyruvate or lactate contribution was plotted based on the percentage of (M + 3) pyruvate or (M + 3) lactate in the total pyruvate or lactate pool, respectively. (PDF 134 KB) [file 40170_2014_136_MOESM4_ESM.pdf]

# Figure S3

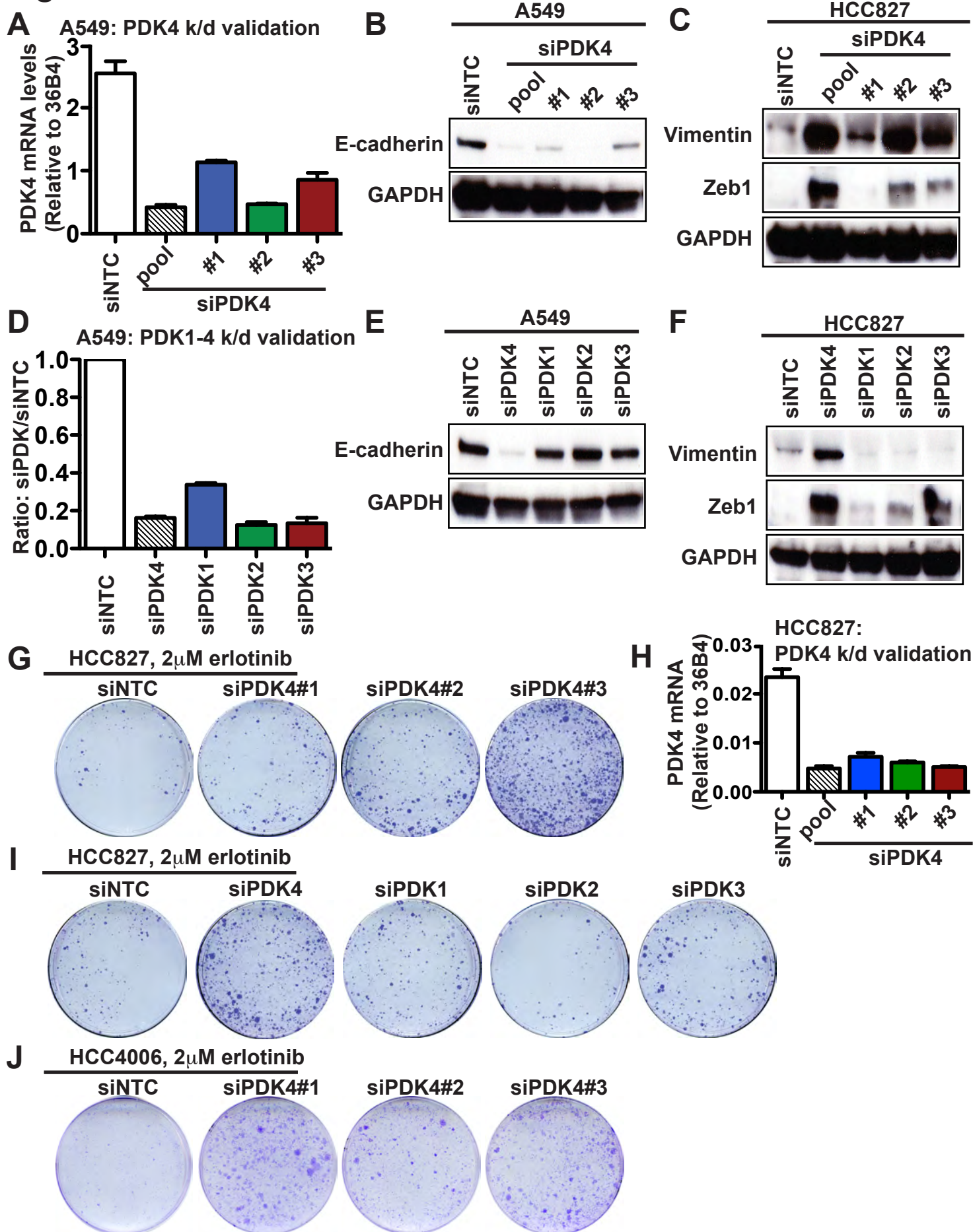

Supplement: Supplementary file 5 — Additional file 5: Figure S3: Deconvolution of the siPDK4 smart pool, and the effects of PDK1-PDK3 knockdown on EMT. (A-C) A549 and HCC827 cells were transfected with siPDK4 pool or three individual siRNAs from the pool at one day and three days post-seeding. Two days after the second transfection, the cells were lysed for immunoblotting and qRT-PCR. (A) PDK4 knockdown (k/d) efficiency from individual siRNAs in A549 cells, evaluated using qRT-PCR. (B) Immunoblots showing the effects of PDK4 knockdown on the epithelial marker E-cadherin in A549 cells, using three individual siRNAs. (C) Immunoblots showing the effects of PDK4 knockdown on mesenchymal markers Vimentin and Zeb1 in HCC827 cells, using three individual siRNAs. (D-F) A549 and HCC827 cells were transfected with siRNA smart pools of siNTC, siPDK1, siPDK2, siPDK3 or siPDK4 at one day and three days post-seeding. (D) Validation of knockdown (k/d) efficiency of each PDK siRNA on the corresponding PDK isoform, quantified by qRT-PCR. The y-axis represents the particular PDK mRNA levels in siPDK-transfected cells over siNTC-transfected cells. (E) Immunoblots showing the effects of each PDK isoform knockdown on the epithelial marker E-cadherin in A549 cells. (F) Immunoblots showing the effects of each individual PDK isoform knockdown on the mesenchymal markers Vimentin and Zeb1 in HCC827 cells. (G) Colony formation capacity of HCC827 cells treated as in C, in the presence of 2 μM erlotinib. (H) PDK4 knockdown (k/d) efficiency using individual siRNAs in HCC827 cells, as evaluated in A. (I) Colony formation capacity of HCC827 cells treated in F, in the presence of 2 μM erlotinib. The siNTC and siPDK4 plates in I are reproduced from Figure 3C to facilitate a direct comparison amongst all parameters. (J) Colony formation capacity of HCC4006 cells treated as in G, in the presence of 2 μM erlotinib. (PDF 210 KB) [file 40170_2014_136_MOESM5_ESM.pdf]

Figure S4

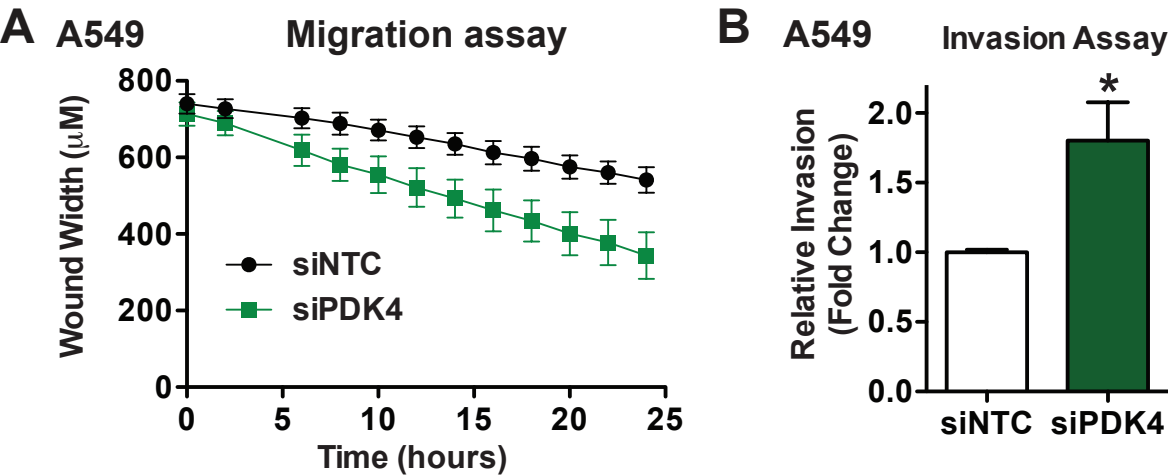

Supplement: Supplementary file 6 — Additional file 6: Figure S4: PDK4 knockdown promotes cell migration and invasion. A549 cells were transfected with siNTC pool#2 or the siPDK4 pool at one day and three days post-seeding. The day after the second transfection, cells were seeded in an IncuCyte ImageLock plate for migration assay (A), and a Boyden chamber for invasion assay (B), as described in the Extended Methods. The migration assay shows the average of 10 wells from one experiment, which is representative of two independent experiments. The invasion assay is the average of two independent experiments each containing two replicates. *, p < 0.05. (PDF 262 KB) [file 40170_2014_136_MOESM6_ESM.pdf]

Figure S5

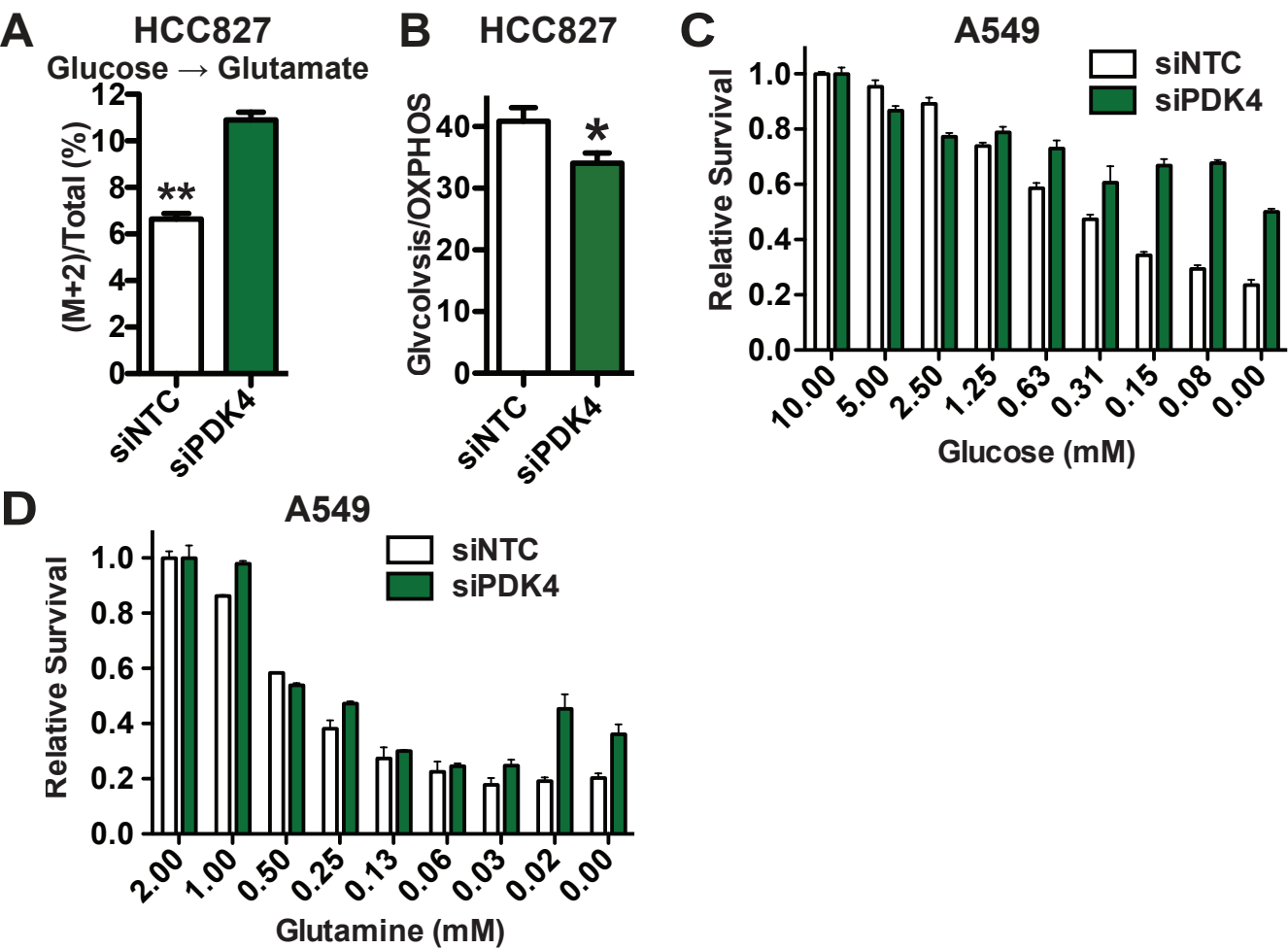

Supplement: Supplementary file 7 — Additional file 7: Figure S5: PDK4 knockdown promotes metabolic rewiring and cell survival under low glucose conditions. HCC827 cells were transfected with siNTC pool#2 or the siPDK4 pool at one day and three days post-seeding. (A) The day after the second transfection, the cells were incubated overnight with growth media containing 13C-U-glucose, and then subjected to LC-MS. Glucose to glutamate contribution was plotted based on the percentage of (M + 2) glutamate in the total glutamate pool. Each data point is from three separate biological samples generated at the same time. (B) The day after the second transfection, the cells were seeded in Seahorse plates to measure Glycolysis/OXPHOS ratio (defined by PPR/OCR). Each data point is from eight wells. (C, D) The day after the second transfection, cells were plated in 96 well plates. After overnight incubation, cells were switched to media containing various concentrations of glucose (C) or glutamine (D). Three days after, cell survival was measured using CellTiterGlo. Each data point is an average of three wells. Data are plotted as mean +/-SEM. *, p < 0.05, **, p < 0.01. (PDF 311 KB) [file 40170_2014_136_MOESM7_ESM.pdf]

Figure S6

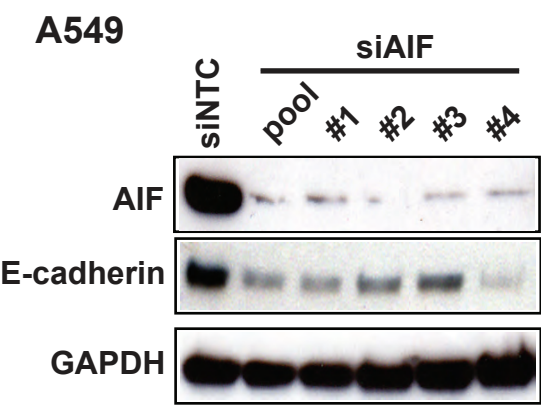

Supplement: Supplementary file 9 — Additional file 9: Figure S6: Deconvolution of the siAIF smart pool. A549 cells were transfected with siNTC pool#2, the siAIF smart pool, or individual siRNAs in the siAIF smart pool, at one day and three days post-seeding, then lysed for immunoblotting. (PDF 437 KB) [file 40170_2014_136_MOESM9_ESM.pdf]
